# Analysing injuries to dancers working in the commercial dance industry

**DOI:** 10.1093/occmed/kqag031

**Published:** 2026-05-23

**Authors:** J A Russell, P J Armour, L L Hodge, R Pereira, S M Petery

**Affiliations:** Department of Athletic Training, Laboratory for Science and Health in Artistic Performance, Ohio University, Athens, OH 45701, USA; Department of Kinesiology, Rice University, Houston, TX 77005, USA; Department of Biosciences, Rice University, Houston, TX 77005, USA; Dancers Network, London, SS2 6AP, UK; Chocho Creative, LLC, Los Angeles, CA 91601, USA; Department of Athletic Training, Laboratory for Science and Health in Artistic Performance, Ohio University, Athens, OH 45701, USA; KBR Wyle—Human Health and Performance, NASA Johnson Space Center, Houston, TX 77058, USA

## Abstract

**Background:**

Commercial dancers work in live shows, films and other venues. They perform many dance genres and undergo substantial physical demands. This occupational community sustains many injuries but may not seek or have access to specialized medical services.

**Aims:**

To determine, in a sample of dancers in the commercial dance industry, the prevalence of musculoskeletal dance-related injuries, the dancers’ accessibility to medical consultation and the dancers’ healthcare-seeking tendencies over 5 years.

**Methods:**

An electronic questionnaire was distributed to commercial dancers via a commercial dance advocacy organization. One hundred twenty dancers (91 females, 29 males) in the commercial dance industry completed this retrospective study. Data were analysed via descriptive statistics. We hypothesized that the 5-year prevalence of injury would exceed 70%, and that healthcare resources are not widely available to the commercial dance community in relation to injury occurrence, body region of injuries and time loss due to injury.

**Results:**

Ninety-one per cent of the participants reported at least one time-loss injury over the 5-year period. The knee, lower back, ankle and hip were injured most often. Ninety-three per cent of the respondents reported dancing with injury-related pain, and 73% visited a physiotherapist/physical therapist or occupational therapist for at least one injury. Forty-three per cent reported their primary reason for not seeking care was they ‘could not afford the care’.

**Conclusions:**

This study suggests that commercial dancers exhibit a high prevalence of injuries, often dance in pain and may be dissuaded from seeking care for their time-loss injuries by a lack of financial and medical resources.

Key learning points

**What is already known about this subject:** 

- Dancers in all dance genres generally experience many dance-related injuries but do not enjoy the specialized healthcare access they need for those injuries.
- Dancers in the commercial dance industry engender broad appeal across the globe, as their work is available and consumed regularly in television, cinema and video formats, thus warranting careful and efficacious attention to their dance-related injuries.

**What this study adds:** 

- Commercial dancers experience a dance-related injury prevalence above 90%.
- Commercial dancers usually are employed with episodic independent contracts that may lead to them feeling they cannot afford appropriate medical care for their dance-related injuries.
- Some in the commercial dance community lack confidence in the patient–practitioner relationship; that relationship may not be established because dancers have not sought needed care; however, affordability of the care is a primary concern.

**What impact this may have on practice or policy:** 

- Our findings that the occupational field of commercial dance is associated with high injury prevalence support the need for advocacy that improves the conditions for these workers financially and in healthcare provision that is specialized for their artistic craft.
- When encountering performing arts patients such as commercial dancers, healthcare practitioners should focus on compassionate care and continuing professional development for the knowledge necessary to manage dance-related injuries.

## Introduction

Professional performing artists, including dancers, often do not enjoy access to occupational healthcare at the same level that other workers do, particularly not specialized healthcare that considers the rigours of their dance physicality. Commercial dance is an increasingly popular dance sub-speciality with global influence. This is largely due to its diverse dance styles, generational appeal and the abundant international availability of performances that feature its dancers (e.g. televised music awards shows, the American football Super Bowl halftime show, advertising, movies). Commercial dance includes many different genres, whilst demanding constant energy and countless hours from performers who move from one individually contracted job to the next as they continuously seek their next employment. A particularly challenging example is the dancers’ work with pop artists on long-term touring contracts. These place enormous demands on dancers’ bodies due to extended travel, frequent international flights, limited recovery time and the accompanying cumulative physical strain.

Unlike most competitive athletes, dancers must conform to specific aesthetic guidelines and choreography. Moreover, injury or undue pain may place a professional dancer’s career at risk [1,2] or result in them being released from a job contract. Alongside the stressors professional dancers endure, they often receive minimal healthcare provision and they typically cannot access performing arts-focused healthcare that matches care offered to sports participants [3]. Most dancers in the commercial industry are self-employed or part-time employed by audition or direct booking; this limits their healthcare options, especially in countries without national healthcare services. Overall, commercial dance is a medically underserved performing art where lack of healthcare may negatively impact performers’ daily lives. Thus, physical demands placed on this population can cause dance-related injuries (DRIs) that often go unaddressed.

In addition, the commercial dance industry has far less regulation compared to institutional settings. Commercial dancers often are asked to perform a wide range of additional skills (including lifts, partner work and acrobatic elements) without the safety infrastructure typically found in other performance environments. Rehearsal schedules can be intense, with limited awareness of training load across multiple rehearsals and concurrent jobs. Moreover, many commercial dancers continue working through minor injuries for fear of missing out on employment, concerns about being perceived as not good enough and the financial liability of unpaid time off or self-funding healthcare.

Reported time-loss injury incidence per 1000 dance hours in professional ballet, the genre for which data are most available, ranges from 1.1 injuries [4] to 4.76 injuries per 1000 hours of dance, with a mean of 2.8–6.4 injuries per dancer per year [5]. Prior investigations suggest that professional modern/contemporary dancers have a time-loss injury rate of 0.16–1.4 [6] injuries per 1000 hours [7]. Injuries in hip-hop dance, a very common genre for commercial dancers, occur with an incidence of 1.16 per 1000 dance hours [8]. The literature supports that dancers are injured most commonly in the lower limb [4,6,7,9]. However, some research suggests that head, neck and back injuries also are frequently sustained [5,9]. Although dancers in other dance genres have been targeted by research efforts, relatively few data are available for professional dancers and there is no known available research pertaining to dancers in the commercial industry.

In professional dance, many DRIs are comparable to injuries of professional athletes. Yet, professional athletes generally have a sophisticated and attentive medical community at their disposal. Despite the high likelihood of injury in dance noted above, previous research indicates that ∼15% of all DRIs in professional ballet and modern dancers are not reported [10]. These must receive appropriate care when one’s occupation and ability to attain a high level of performance depend on their physical abilities being intact. Therefore, the aims of this study were to determine in a sample of dancers in the commercial dance industry the prevalence of musculoskeletal DRIs, the dancers’ willingness to access medical consultation and the dancers’ healthcare-seeking tendencies over 5 years. We hypothesized that the 5-year injury prevalence of at least one injury would be 70%, that less than half the dancers would access medical consultation, and that there would be barriers to the dancers obtaining high-quality healthcare for their injuries.

## Methods

Participants were recruited through an international advocacy organization that works to promote and protect the rights of dancers in the commercial industry. This entity was founded by two of our co-authors, who additionally served in this study as community-engaged co-investigators [11,12]. Their 598 members were alerted to the study. Word of mouth, email and social media were all utilized to communicate necessary recruitment information to potential participants; for maximum reach, the members were not restricted from inviting commercial dancers they knew outside the organization. The study received ethical approval from our university’s Institutional Review Board, and all participants gave their informed consent prior to beginning the survey. Participants who did not identify as a professional commercial dancer, were not 18 years of age or older or who failed to provide any data beyond basic demographics were excluded from the study’s analysis. Secondary to the exploratory nature of the study, there were no other exclusion criteria.

The electronic Qualtrics survey (Qualtrics, Inc., Provo, UT, USA) gathered from the dancers their demographic information, musculoskeletal DRI history and healthcare consultation experiences for their injuries. The demographics portion included biological sex, age, years of total dance experience and years of experience dancing professionally. Following the demographic questions, participants completed a questionnaire that was developed from a similar study involving university dancers [13]. The questions inquired about the participants’ musculoskeletal DRI history over a 5-year period from January 2016 to January 2021.

Whilst self-reported recall injury data may be inherently inaccurate, those inaccuracies likely underestimate true injury occurrence [14]. Because we were interested in the number of dancers who reported at least one injury, the underestimation possibility was accepted. Although the recall period spanned the COVID-19 pandemic, the dancers were in varying stages of employment during the pandemic. Thus, trying to make the survey’s questions account for the presence of the pandemic would have unnecessarily complicated the dancers’ ability to provide accurate answers.

The questions included quantity and body location of injuries, willingness to dance with pain and medical advice sought. Answer formats were both fill-in-the-blank and multiple-choice, depending on the question being asked. Where necessary, questions included an ‘Other’ portion in which participants were able to detail unique information or factors they deemed important to their injury experiences. This definition of DRIs, adapted from Air *et al.* [15], was provided to the dancers: ‘A dance-related injury is any neuromusculoskeletal condition sustained as the result of dancing activity that causes a dancer to stop or modify their dancing’. However, to provide the participants with a concrete prompt for their responses about injury occurrences, we worded our questions to enquire about any injuries that ‘caused you to miss one or more classes, rehearsals or performances’, i.e. a time-loss injury based on missing at least one dance event [4,16,17].

Understanding the nature of the participants’ dance injuries and their reasons for not obtaining medical care was of prime importance. Answer choices for avoiding healthcare consultation spanned fear, lack of financial means, distrust of medical providers, lack of time availability, prior bad experience with healthcare and knowledge of injury self-care.

We analysed the data using frequency counts and descriptive statistics to obtain an overall picture of how our sample of dancers in the commercial industry responded to DRIs and what their experiences have been with accessing healthcare for those injuries.

## Results

**Figure 1:**
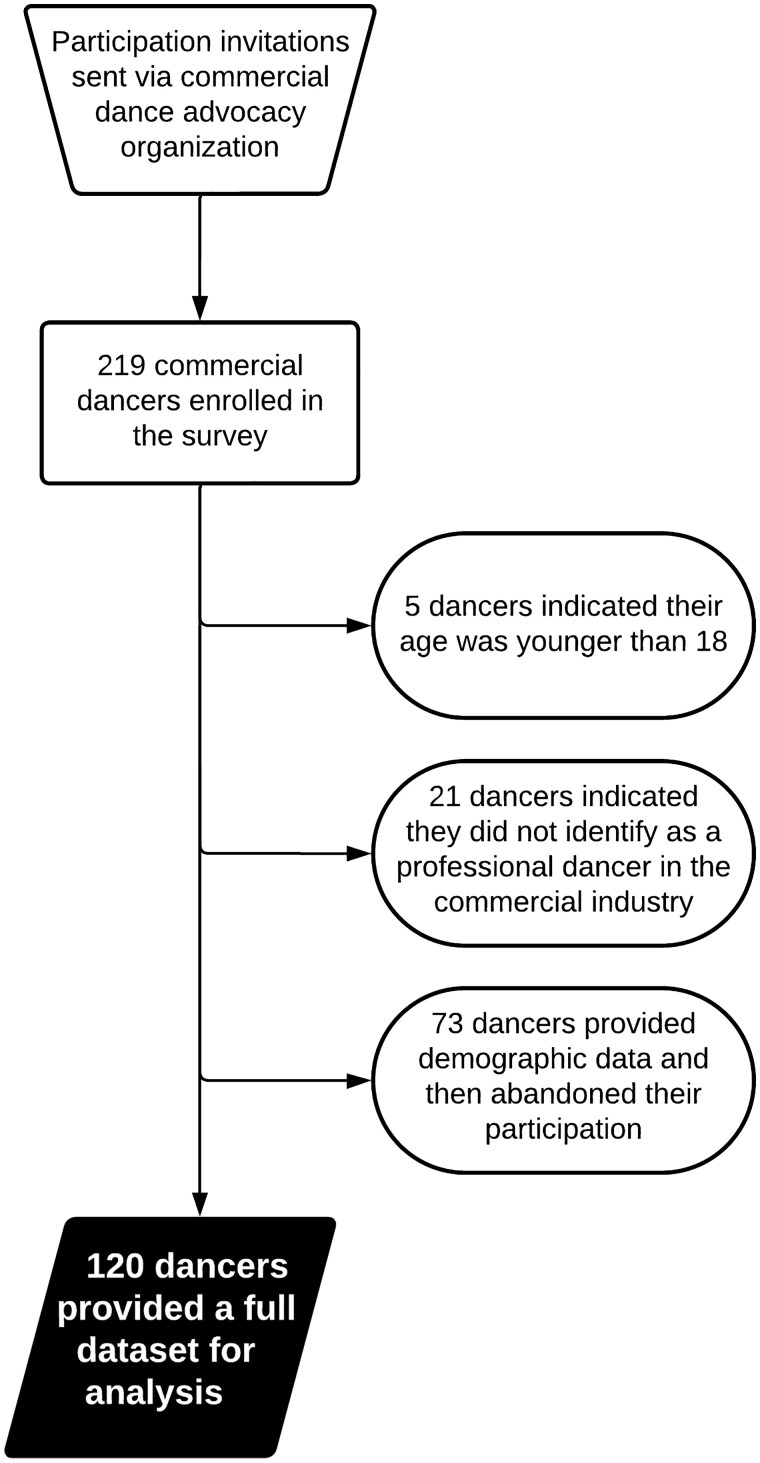
Flowchart showing the progression of recruitment and engagement of participants.

Of the 219 individuals who responded to the survey invitation, 120 provided all data or declined to answer only one question; the remainder were not included in our study because they did not meet the inclusion criteria for age or commercial dance participation, or because they exited the survey without providing answers beyond their demographics (see Figure 1). The mean age of these professionals was 25.4 ± 4.5 years (range = 18–40 years), with 29 males and 91 females rounding out our composite. Participants reported dancing an average of 20.5 ± 16.1 hours per week. The top three geographical regions where they worked as dancers were the UK (60%, *n* = 72), continental Europe (22%, *n* = 26) and the USA (14%, *n* = 17). Eighty-eight per cent (*n* = 106) of the dancers indicated they participate in hip-hop dance. This was followed by jazz (57%, *n* = 68), modern/contemporary (42%, *n* = 50) and musical theatre (32%, *n* = 38). Full demographic data are shown in Table 1.

**Table 1:** Demographic data of dancer survey respondents (*n* = 120; 91 F, 29 M)

	Age (yrs)	Dance Hours per Week^a^	
**Mean**	25.4	20.5	
**± SD**	4.5	16.1	
**Years of Dance Experience**		**Work Location^b^**	**Regular Dance Genres (individuals could select all that applied)**
**0–5 yrs:** 9 (8%)		**UK:**72 (60%)	**Hip-Hop:** 106 (88%)
**5–10 yrs:**23 (19%)		**Other Europe:**26 (22%)	**Jazz:** 68 (57%)
**10–15 yrs:** 32 (27%)		**USA: ** 17 (14%)	**Modern/Contemporary:**50 (42%)
**15–20 yrs:** 29 (24%)		**Canada:** 2 (2%)	**Musical Theatre:**38 (32%)
**20+ yrs:**27 (23%)		**Asia:** 2 (2%)	**Ballet:** 27 (23%)
			**Tap:** 15 (13%)
			**World (Ethnic) Dance:** 4 (3%)
			**Ballroom:**1 (1%)
			**Other:** 40 (33%)

a Three dancers did not report hours of dance per week.

b One dancer did not report work location.

Considering DRIs that caused individuals to miss one or more classes, rehearsals or performances in the 5-year window of the study, 9% of the participants (*n* = 11) had not experienced in the last 5 years any neuromusculoskeletal condition sustained as the result of dancing activity that caused them to miss one or more classes, rehearsals or performances; 40% (*n* = 48) reported 1–2 injuries; 34% (*n* = 41) reported 3–4 injuries; whilst 17% (*n* = 20) had experienced 5 or more such injuries. Thus, the 5-year prevalence of at least one DRI was 91%, and the incidence per dancer in that 5 years was 3.3 injuries. When asked about dancing with pain they felt could be indicative of an injury, 76% (*n* = 91) responded that either they had done so previously and would do so again, or they had not previously done so but would do so in the future. The remaining respondents reported that they had never done this or had done so previously but would not do so in the future.

The frequency distribution of injured body regions for all survey respondents is presented in Table 2. There were 362 responses to ‘if you have experienced one or more dance-related injuries that caused you to miss one or more classes, rehearsals or performances, where was/were the injury(ies) located? (please check all that apply)’. The injured region reported by the greatest number of dancers was the knee (53%, *n* = 64), followed by the lower back (45%, *n* = 54), the ankle (33%, *n* = 40) and the hip (30%, *n* = 36). Overall, lower extremity DRIs represented 59% (*n* = 214) of the total reported injuries; head, neck and back injuries were 29% (*n* = 103); upper extremity injuries were 8% (*n* = 30); rib injuries 1% (*n* = 5) and injuries to ‘other’ locations were 3% (*n* = 10) of the total injuries.

**Table 2:** Frequency distribution of injured body region (*n* = 120 dancers)

**Injury location** ^a^	Frequency	% of total injuries	% of dancers reporting	
**Hip**	36	10%	30%	Lower extremity: 214 injuries (59% of total injuries)
**Groin**	18	5	15	
**Thigh**	11	3	9	
**Knee**	64	18	53	
**Shin**	14	4	12	
**Calf**	6	2	5	
**Ankle**	40	11	33	
**Foot**	14	4	12	
**Toe**	11	3	9	
**Head**	2	1	2	Head, neck, rib cage and back: 108 injuries (30% of total injuries)
**Neck**	25	7	21	
**Rib cage**	5	1	4	
**Upper/mid back**	22	6	18	
**Lower back**	54	15	45	
**Shoulder**	21	6	18	Upper extremity: 30 injuries (8% of total injuries)
**Arm**	3	1	3	
**Hand or finger**	6	2	5	
**Other**	10	3	8	10 injuries (3% of total injuries)
**Total injuries reported**	362			362 (100%)

a Dancers could select all that applied.

Concerning the participants’ responses when asked about their experience with DRIs severe enough to seek medical attention (‘How many times in the last 5 years have you experienced a dance-related injury severe enough to require you to seek the advice of a medical professional?’), 27% (*n* = 32) identified 1, 30% (*n* = 36) divulged 2 and 16% (*n* = 19) indicated 3 such injuries. Fifteen per cent (*n* = 18) of participants reported four or more medical consultation DRIs. Whereas ∼13% (*n* = 15) of the dancers reported they had not experienced a DRI severe enough to consult a healthcare professional, the 5-year prevalence of medical consultation DRIs was 87%.

As shown in Table 3, 7 out of 10 dancers (73%, *n* = 88) who consulted a healthcare professional elected to visit a physical therapist/physiotherapist (physio) or occupational therapist when they sustained a DRI, followed by chiropractor, acupuncturist or massage therapist (53%, *n* = 63) and doctor of medicine (MD) or doctor of osteopathy (DO) (45%, *n* = 54). Many respondents elected to seek medical advice about one or more of their injuries from someone other than a licensed healthcare practitioner, most commonly a dance teacher (32%, *n* = 38).

**Table 3:** Frequency distribution of healthcare professional consultations reported (120 respondents reporting)

Person approached for healthcare consultation^a^	Dancers reporting (%)
**I have not consulted a healthcare professional regarding my dance-related injury(ies)**	8 (7)
**Physical therapist/physio or occupational therapist**	88 (73)
**Chiropractor, acupuncturist, massage therapist**	63 (53)
**Medical doctor (MD) or doctor of osteopathy (DO)** ^b^	54 (45)
**Dance teacher**	38 (32)
**Athletic trainer**	19 (16)
**Exercise practitioner (e.g. pilates instructor, personal trainer)**	19 (16)
**Nurse, nurse practitioner (CNP) or physician assistant (PA)**	7 (6)
**Herbalist or homeopathic or naturopathic practitioner**	6 (5)
**Reflexologist**	4 (3)
**Other**	3 (3)

a Dancers could select all that applied.

b Doctor of osteopathy is an MD-equivalent medical degree in the USA, not to be confused with a non-physician osteopath in other countries.

Of 94 dancers who indicated they did not seek medical attention for at least one of their DRIs (Table 4), the most common reason of the eight possible survey answers for not seeking care was that they ‘could not afford the care’ (43%, *n* = 40), followed by ‘knew how to care for the injury myself’ (33%, *n* = 31), ‘injury not severe enough’ (28%, *n* = 26) and ‘did not expect healthcare provider to give helpful care or advice’ (27%, *n* = 25). Twenty-six of the dancers did not provide answers to this question.

**Table 4:** Frequency distribution of reasons for not seeking healthcare for a DRI (94 dancers reporting; 26 dancers did not provide an answer)

Reason for not seeking care^a^	Dancers reporting (%)
**Could not afford the care**	40 (43)
**Knew how to care for the injury myself**	31 (33)
**Injury not severe enough**	26 (28)
**Did not expect healthcare provider to give helpful care or advice**	25 (27)
**Did not have time to go**	18 (19)
**Was afraid to go**	18 (19)
**Did not know the best provider to address the injury**	14 (15)
**Had a previous bad experience with a healthcare provider**	10 (11)
**Have not experienced an injury**	4 (4)

a Dancers could select all that applied.

## Discussion

Our descriptive study provides evidence of a high prevalence of DRI and good healthcare seeking in commercial dancers, with concomitant barriers to obtaining healthcare in this understudied population.

Specifically, our hypothesis that the 5-year injury prevalence of at least one DRI would be 70% was supported; we calculated a prevalence of 91%. We predicted that fewer than half the dancers would access medical consultation; however, most (93%) did report a willingness to consult with a healthcare provider. Our hypothesis about their barriers to obtaining specialized care for their DRIs was correct, as they expressed a lack of resources to obtain care and distrust of the providers.

The DRI prevalence in our participants is higher than the 67% prevalence over 15 years for professional modern dancers [18] and the ∼70% prevalence for professional ballet dancers [19]. Whilst, to our knowledge, injury data on commercial dancers are not available elsewhere, the literature generally supports that dancers in a variety of genres are most commonly injured in the lower limb [18–20]; our data suggest similarly. More specifically, however, our participants’ substantial number of injuries to the head, neck and back are likely accounted for by the variety of dance genre expertise required of dancers in the commercial industry. This may place greater than typical stress on the axial skeleton—especially in view of 88% of our respondents indicating participation in hip-hop. For comparison, prior hip-hop data [21] depict the regions most commonly injured: shoulder, neck, lower back and upper back. Whilst we did not enquire about which injuries occurred during specific genres, we note this characteristic of our results that relates well to previous research.

Seventy-six per cent of our participants had danced with pain they felt indicated a DRI and expressed their intention to continue to do so in the future or, at least, an intention to dance through such pain if they had not experienced it in the past but encountered it in the future. This is consistent with literature suggesting that dancers exhibit a ‘dance through pain’ coping mentality [3,20,22,23].

When our participants did not seek healthcare for a DRI, more than one-third revealed feeling they would not get helpful advice from a medical professional or they had an untoward previous encounter; these confirm prior research [20]. Unfortunately, the lack of compassion and understanding of DRIs by healthcare practitioners has led to a breakdown in trust between the dance and medical communities [3,15,20].

We found it interesting that one-third of respondents reported their inability to afford healthcare for DRIs. The USA, where just 14% of our participants worked, is the only employment locale of our participants without some form of national healthcare provision. In trying to reconcile the responses about the affordability of care, we assume that many dancers in national healthcare countries were not able to access care in a timely fashion without having money for more expedient private care, as excessive wait times are a common point of patient dissatisfaction in these nations [24,25].

About one-third of the dancers consulted a dance teacher about their DRIs. Logically, dancers and their teachers enjoy a strong relationship. The educators usually are neither licensed nor qualified to administer healthcare and should be discouraged from doing so [20]. Nonetheless, it can be inferred that with little financial support, commercial dancers struggle to find injury care and thus confer with those closest or most available to them who do not charge fees for such consultations.

Our study has limitations, with recall bias chief amongst these. During the COVID-19 pandemic, the commercial dance industry did not return to normal for ∼18 months. However, the dancers were necessarily working to a greater extent than most other occupations because of their significant role in the media for fields such as advertising. After considering alternatives, we elected to ask participants for 5 years of self-reported DRI data that included their work during the COVID-19 timeframe. We acknowledge that both the length of recall time and the pandemic may have affected their recall, as injury recall is better for more severe injuries than less severe injuries [26] and worsens as recall time increases [26,27], including for occupational injuries [28]. However, we therefore believe the injury data we collected underestimates the true injury prevalence. That is, dancers in the commercial industry likely sustain more injuries than our survey identified.

We also could not calculate a response rate for our participants. We started with 598 dancers in the international dance advocacy network, but the nature of how the commercial dance industry operates required us to recruit through their further connections via email, social media and word-of-mouth. Whilst not allowing response rate calculation, this was our most practical recruitment option.

Finally, to our knowledge, most dancers in the commercial industry have hitherto not participated in research of this type. Respondents may have had difficulty with some questions, thus potentially impacting results. Throughout data collection, many surveys were started but not completed. We surmise that lack of survey experience and the dancers’ ultra-busy lives may have played a role in survey incompletions. Moreover, commercial dancers typically are self-employed, freelance professionals hired on short-term contracts for specific projects. Their job security often is volatile, a stressor that may have influenced their decisions around completing injury reporting portions of the survey.

We provide evidence that dancers in the commercial industry have a high prevalence of DRIs, and, with uncertainty of contracted financial support, they often feel unable to afford proper medical consultation. Clinicians should consider these findings when working with dancers. This population is medically underserved, and the patient–clinician trust relationship is not satisfactory. Rebuilding this trust can occur through specialized education of clinicians about the intricacies of this dance community and compassionate provision of care [3].

This article summarizes musculoskeletal DRIs in a sample of dancers in the commercial industry and illustrates challenges these dancers face when injured. We have established a foundation for better clinical care and further research relative to injury occurrence, body region, time loss and healthcare provision.

## Data Availability

This study's dataset will be provided upon reasonable request made to the corresponding author.
